# Trematode infections in cattle in Arumeru District, Tanzania are associated with irrigation

**DOI:** 10.1186/1756-3305-7-107

**Published:** 2014-03-20

**Authors:** Jahashi Nzalawahe, Ayub A Kassuku, J Russell Stothard, Gerald C Coles, Mark C Eisler

**Affiliations:** 1 Department of Veterinary Microbiology and Parasitology, Sokoine University of Agriculture, P.O. Box 3019, Morogoro, Tanzania; 2 Liverpool School of Tropical Medicine, Pembroke Place, Liverpool L3 5QA, UK; 3 School of Veterinary Sciences and Cabot Institute, University of Bristol, Langford House, Langford, Bristol BS40 5DU, UK

**Keywords:** Cattle, *Fasciola*, Food security, Paramphistome, Trematode, *Schistosoma*, Sub-Saharan Africa

## Abstract

**Background:**

The relationship between the environment and infection of cattle with trematodes was studied at Arumeru District, Arusha Region, northern Tanzania. Randomly selected villages were grouped into three cattle management strata, (i) zero-grazing (ZZ) (ii) communal grazing without irrigation (ZC) and (iii) communal grazing with irrigation (ZCI).

**Methods:**

Faecal samples were collected from 241 cattle, and processed using the Flukefinder® method. Snail intermediate hosts were collected with a snail scoop from the water bodies in the study villages and identified morphologically.

**Results:**

The overall prevalence of *F. gigantica*, paramphistomes and *S. bovis* were 33%, 37% and 2% respectively. Prevalence for *F. gigantica*, paramphistomes, and *S. bovis* for each stratum were, zero-grazing (ZZ) (29.7%, 36.0% and 0%), communal grazing without irrigation (ZC) (6.3%, 15.0% and 3.8%) and communal grazing with irrigation (ZCI) (57.7%, 56.7% and 1.0%) respectively. The differences between strata were significant for *F. gigantica* (p < 0.001) and paramphistomes (p < 0.05) but not for *S. bovis*. Irrigation could account for the high prevalence of *F. gigantica* and paramphistomes in the ZCI stratum as compared to the ZZ and ZC strata. The higher prevalences of *F. gigantica* and paramphistomes in the ZZ stratum compared with the ZC stratum were unexpected and attributed to the practice of farmers in some ZZ stratum villages buying fodder for their cattle obtained from pastures in ZCI villages.

**Conclusion:**

Trematode infections in cattle are prevalent in Arumeru District. *Fasciola gigantica* and paramphistomes are associated with grazing in areas with irrigation of crops. Zero-grazing of cattle does not necessarily prevent the risk of infection.

## Background

Trematode infections in cattle especially *Fasciola gigantica* cause severe economic losses in sub-Saharan African countries [1-3], including Tanzania [4]. Trematode species that have been reported in Tanzania are *F. gigantica*[5], *Fasciola hepatica*[6], *Schistosoma bovis*[7], *Paramphistomum* species and *Dicrocoelium hospes*[8]. Abattoir surveys [9-13], found trematode infections in the southern highlands zone, northern zone, Lake zone and eastern zone of Tanzania and the epidemiology and control of trematode infections in cattle has been studied in the Southern Highlands [5-7,14]. Scanty information is available on the epidemiology in cattle in other parts of the country where trematode infections have been reported. This study was conducted in Arumeru District in northern Tanzania to investigate the epidemiology of trematode infections in cattle under different cattle management systems in order to generate base line data that will provide information for the design of effective control strategies.

## Methods

### Study design and sample size

A cross-sectional study was conducted from August to September 2012 to determine the prevalence of trematode infections in cattle and to identify the potential snail intermediate hosts in Arumeru District, Arusha Region, Tanzania. Study villages were selected using probability proportional to size, based on their cattle populations as reported by the local District Veterinary Office. A total of 15 study villages were selected and further divided into three strata according to their cattle management system and whether there was irrigation of crops. Generally in the wet season all villages confined cattle to avoid their consumption of growing crops and practiced zero-grazing. The three strata comprised (i) villages which confine cattle and practice zero-grazing all year-round (ZZ), (ii) those without irrigation which practice communal grazing in the dry season (ZC) and those with irrigation practicing communal grazing in the dry season (ZCI). Sample size was calculated using Bennett’s formula for cluster sample surveys to enable estimation of prevalence with ±5% absolute precision and 95% certainty [15].

### Parasitological examination of cattle

A total of 241 cattle were selected and faecal samples were collected per rectum, using sterile surgical gloves that were then turned inside out to contain the sample, and labelled with a unique identification number encoding area, age (categorized as weaners 6–24 months and adult above 24 months) sex, date, herd identity and sample number before storing them in an ice packed cool box awaiting examination. Faecal samples were processed using a Flukefinder® (Richard Dixon, ID, US) in accordance with the manufacturer’s instructions. Flukefinder® is a kit comprising a unit made up of two 50 mm wide sieves of approximately 125 nm and 30 nm mesh. Briefly 2 g of faeces were mixed with water then poured into the Flukefinder® unit and flushed well with water. Larger faecal debris were retained by the larger diameter sieve and discarded. Faecal material retained in the smaller diameter sieve, including trematode eggs, was back washed into a 50 ml plastic cup. This was allowed to settle for five minutes then the supernatant was poured off and the remaining faecal material poured into a 50 mm petri dish. Water was added to fill the petri dish, and then the contents allowed to settle for 30 seconds before the supernatant was poured off. Three drops of methylene blue were added to the remaining faecal material which was then examined under a x4 objective stereoscopic dissecting microscope. Eggs were identified using key morphological features [16] and counted. All work on animals described in this manuscript was conducted in accordance with internationally recognised guidelines and approved by Sokoine University of Agriculture.

### Snail collection

Water bodies were visited for collection of snail intermediate hosts in each study village that included: rivers, irrigation canals, swamps and water reservoirs. Collection of snails was performed by scooping [17,18] and or hand picking conducted by two people at each site for 20 min. Snails were identified morphologically using a field guide to African freshwater snails [19,20]. Furthermore, each of the snails was placed in a 10 ml beaker filled with 6 ml of distilled water and exposed to light overnight to stimulate shedding of the cercariae. The following morning water in each beaker was poured into the petri dish, and examined under the x4 objective stereoscopic microscope for the presence of cercariae.

### Data analysis

Prevalence was calculated as the number of positive faecal samples over the total number of faecal samples examined at district, management, village and age strata levels. Differences in prevalence were assessed with generalised linear models with binomial errors using R- statistical software (version i386 2.15.0; the R Foundation for Statistical Computing, http://www.R-project.org). Fisher’s exact test was used for significance of differences between the three strata in terms of age profiles of cattle, because of the expected value (4.2) for the youngest age group in the ZZ stratum was less than 5.

## Results

### Prevalence of trematode infections in cattle

*Fasciola gigantica*, paramphistome and *Schistosoma bovis* infections were found in all but two of the fifteen villages in Arumeru District in which cattle were examined (Table 1). The overall prevalences of *Fasciola*, paramphistome and *Schistosoma* infections in 241 cattle were 33.2%, 37.3% and 1.7% respectively. The prevalences of these three genera of trematodes in cattle in each stratum are shown in Table 2.

**Table 1:** Trematode infections in cattle in study villages in Arumeru District, Tanzania

**Management Stratum**	**Village**	**n**	***Fasciola gigantica***	**Paramphistome**	***Schistosoma bovis***
ZZ	Sing'isi	16	11 (69%)	15 (94%)	0 (0%)
	Patandi	16	8 (50%)	8 (50%)	0 (0%)
	Ngyani	16	0 (0%)	0 (0%)	0 (0%)
	Ndoombo	16	0 (0%)	0 (0%)	0 (0%)
ZC	Samaria	16	2 (13%)	3 (19%)	1 (6%)
	Kolila	16	2 (13%)	2 (13%)	0 (0%)
	Kitefu	16	1 (6%)	2 (13%)	2 (13%)
	Shishitony	16	0 (0%)	2( 13%)	0 (0%)
	Leguruki	16	0 (0%)	3 (19%)	0 (0%)
ZCI	Lekitatu	15	14 (93%)	100 (15%)	1 (7%)
	Makiba	18	15 (83%)	13 (72%)	0 (0%)
	Usa River	16	12 (75%)	12 (75%)	0 (0%)
	Uwiro	16	6 (38%)	7 (44%)	0 (0%)
	Mikungani	16	5 (31%)	4 (25%)	0 (0%)
	Olkung'wado	16	4 (25%)	4 (25%)	0 (0%)

ZZ: Zero-grazing, ZC: Communal grazing without irrigation, ZCI: Communal grazing with irrigation.

**Table 2:** Trematode infections in cattle in each management stratum in Arumeru District, Tanzania

**Stratum**	**Parasite**	**Infected**	**Prevalence**	**C.I. 95%**
ZZ	*Fasciola gigantica*	19	29.7%	11.1 – 58.7%
(n = 64)	Paramphistome	23	35.9%	13.8 – 66.4%
	*Schistosoma bovis*	0	0.0%	ND
ZC	*Fasciola gigantica*	5	6.3%	0.9 – 34.1%
(n = 80)	Paramphistome	12	15.0%	3.7 – 44.4%
	*Schistosoma bovis*	4	5.0%	ND
ZCI	*Fasciola gigantica*	56	57.7%	35.4 – 77.3%
(n = 97)	Paramphistome	55	56.7%	32.7 – 77.8%
	*Schistosoma bovis*	1	1.0%	ND

ZZ: Zero-grazing, ZC: Communal grazing without irrigation, ZCI: Communal grazing with irrigation.

The differences in prevalence of *F. gigantica* were investigated using a generalised linear model with a quasibinomial empirical scale parameter to allow for overdispersion. Differences among strata were significant (p < 0.02), with stratum ZCI prevalence significantly greater than stratum ZC, (but neither significantly different to stratum ZZ). Compared with cattle zero-grazing all year round, prevalence of *F. gigantica* in cattle grazing communally during the dry season was not significantly different (p > 0.1). Cattle in villages practicing irrigation had significantly higher prevalence (57.7%) than in those that did not (16.7%). The odds ratio for risk of *F. gigantica* infection associated with irrigation was 6.8 (95% C.I. 1.45 – 32.0, p < 0.05), corresponding to a relative risk of 3.5 (C.I. 95% 1.3 – 9.8). Differences among management strata in prevalence of paramphistome infection were similar to those for *Fasciola*, but not significant (p = 0.097) and nor was the practice of irrigation (p = 0084). Numbers of cattle with *Schistosoma* infection were too few for meaningful statistical analysis; three cattle were infected (in two villages) in stratum ZC and one in stratum ZCI, but none in stratum ZZ.

There was no significant difference between the three strata in terms of age profiles of cattle (p = 0.38). Adult cattle had the highest prevalence of *F. gigantica* and paramphistomes while weaners had the highest prevalence of *S. bovis* (Table 3). The differences of prevalence between age groups were statistically significant for paramphistomes (p > 0.05) but not for *F. gigantica* or *S. bovis*. A total of 96 (40%) out of 241 cattle had co-infections of *F. gigantica* and paramphistomes, villages under ZCI stratum having the most co-infection compared to other villages. The faecal egg counts for *F. gigantica*, paramphistomes and *S. bovis* ranged from 0–167, 0–483 and 0–1 eggs per gram (epg) respectively. The majority of cattle had fewer than 10 epg.

**Table 3:** Prevalence of trematode infections in cattle of each age group in Arumeru District, Tanzania

	**Weaners**	**Adults**
	**(6–24 months)**	**(> 24 months)**
*Fasciola gigantica*	(26.5%)	(35.8%)
Paramphistome	(26.5%)	(41.6%)
*Schistosoma bovis*	(3.4%)	(1.7%)

### Trematode infection in snails

A total of 761 snails were collected including 215 *Lymnaea* (*Radix*) *natalensis*, 545 *Biomphalaria* species and 1 *Bulinus tropicus*. The majority of the snails were collected in irrigation canals and rice fields in villages with irrigation, with the exception of 11 *L. natalensis* that were collected in one swampy location at a zero-grazing village (Ngyani). No snails were collected in villages that practiced communal grazing without irrigation. Seven *L. natalensis* were found to shed *Xiphidiocercariae* (not of veterinary importance).

## Discussion

On the basis of faecal egg counts, cattle in Arumeru District were found to be infected with three genera of cattle trematodes with overall prevalences of 33%, 37% and 2% for *F. gigantica*, paramphistomes and *S. bovis* respectively. While these figures are lower than those in previous studies in the southern highlands of Tanzania, which reported prevalences of 58.5%, 75.2% and 22.8% respectively for the three genera of trematode [13,14,21], prevalences in individual villages varied greatly, with 90-100% of tested cattle infected with *F. gigantica* and paramphistomes and up to 13% with *S. bovis* in some villages (Table 1). The differences in prevalence might be due to the variations in ecological and climatic conditions in the study areas [22,23]. Unsurprisingly, the prevalences of *F. gigantica* and paramphistomes were higher in adult cattle than in weaners, reflecting their greater length of exposure to infection [5,24,25]. The prevalence of *S. bovis* was higher in weaners than in adult cattle; this was also in agreement with other studies [7]. The lower infection rate in adult animals is probably associated with acquired immunity, which leads to resistance to re-infection [26]. The higher prevalences of *F. gigantica* and paramphistomes in the ZCI stratum might be attributed to irrigation, which provides favourable ecological conditions for growth of intermediate hosts snails and development of trematode larval stages [24,27]. Snail intermediate hosts (*L. natalensis*) of *F. gigantica* were indeed mainly found in the irrigated villages. A high proportion of infected snails in Tanzania is reported to occur at the transition from the rainy season to the dry season [7], and a high proportion of cattle acquire trematode infections at the end of the rainy season, reaching a peak during the dry season [21]. Cattle grazing in the irrigation canals and associated swampy areas in the ZCI stratum during the dry season, are more likely to be highly infected with *F. gigantica* and paramphistomes than cattle in the non-irrigated ZC stratum which graze dryer pasture during the dry season [28,29]. Better understanding of the transmission dynamics of *F. gigantica* through intermediate host snails in the irrigated areas could present opportunities for control. For instance, as *L. natalensis* survives poorly through periods of desiccation [19], cessation of irrigation for an appropriate period during the dry season, perhaps following crop harvest, might be helpful in reducing intermediate host population size.

There was considerable variation in prevalence of *F. gigantica* and paramphistomes among villages within strata (Table 1), especially those in the ZCI and ZZ strata. For both *F. gigantica* and paramphistomes, irrigated villages (ZCI stratum) fell into two groups, those with prevalences greater than 75% (Lekitatu, Makiba and Usa River), and those with prevalences less than 45% (Uwiru, Mikungani and Olkung’wado). It was noteworthy that irrigation in the high prevalence villages was much more extensive than in those with low prevalences. Unexpectedly, *F. gigantica* and paramphistomes were observed in cattle in two of the year round zero-grazed villages (ZZ stratum), Sing’isi and Patandi, which had prevalences of at least 50% for both trematodes. Trematode prevalences in these two villages (50% - 94%) greatly exceeded those in cattle in communally grazed cattle in the ZC stratum villages (0% - 19%), which might have been expected to be at greater risk than zero-grazed cattle [29]. Again, it was noteworthy that farmers in Sing’isi and Patandi acquired cattle fodder from irrigation canals and swamps in Lekitatu village (ZCI stratum), which has extensive irrigation, and hence is likely to be contaminated with the infective metacercaria stage of *Fasciola* and *Paramphistomum* species. Selecting fodder from other sources might provide an opportunity for control of trematode infections in zero-grazed cattle in this region, and farmers in these villages could be educated on the importance of avoiding cattle fodder sourced from contaminated areas, and they might consider growing their own pastures to ensure safe feed for their cattle. Since *L. natalensis* was shown to occur in a swampy area within a zero-grazed village, care should be taken to prevent access to such areas by unconfined, small ruminants, which may also potentially host *F. gigantica*.

The overall prevalence of *S. bovis* infection in this study (2%) was very low compared to other trematodes, and infection was observed in only a few cattle in the ZC and ZCI strata. *Bulinus africanus* and *Bulinus forskalli*, the primary intermediate hosts of *S. bovis*, were not found in the study area. Rather *B. tropicus*, which becomes susceptible to *S. bovis* when previously infected with *Paramphistomum* species [30], was present and this might lead to limited transmission of *S. bovis* and hence the low level of infection observed. Moreover, adult cattle develop acquired immunity expressed as reduction of the fecundity of the female worms and resulting in reduced faecal egg counts [26]. This might lead to some infected animals to be missed by the Flukefinder® method due to very low numbers of eggs in the faecal material. The presence of *S. bovis* in more cattle in communal grazing areas without irrigation (ZC stratum) than with irrigation (ZCI) was unexpected, but the numbers of infected animals were too few to be of significance. Absence of *S. bovis* from zero –grazing cattle (ZZ stratum) could be explained by the mode of transmission of *S. bovis* through cercarial skin penetration when cattle enter contaminated water bodies. This clearly cannot happen with zero-grazed animals.

## Conclusion

This study has established that trematode infections including the major pathogen *F. gigantica* are widely prevalent in cattle in Arumeru District, an important livestock farming area of Tanzania. Infection rates with *F. gigantica* and paramphistomes were greatest in villages practicing irrigation of crops which appeared to increase transmission during the dry season when cattle graze freely. Intermediate host snails were also found mainly in villages practicing irrigation. Further studies on snail dynamics and infection rates in irrigated areas would be useful to inform control strategies. Some villages practicing year round zero-grazing also had high levels of trematode infection, which could be controlled by more careful sourcing of cattle fodder. *Schistosoma bovis* was also detected in small numbers of cattle in a few villages where cattle grazed during the dry season. Finally, in the broader context of food security, it is interesting to note how two common practices intended to make a positive contribution to agricultural productivity in this area, namely irrigation of arable crops and zero-grazing of cattle (primarily as a means of controlling ticks and tick-borne disease) may also have negative impacts on cattle health and productivity by increasing burdens of the clinically important parasite *F. gigantica*. This finding underlines the value of transdisciplinary research and intersectoral collaboration to agricultural development activities in sub-Saharan Africa.

## Competing interests

The authors declare that they have no competing interests.

## Authors’ contributions

This work will form part of the PhD thesis of JN. The study was designed by JN, AK, GC and ME. Field and laboratory work was carried out by JN, AK with guidance from JS, GC and ME. Data analysis was undertaken by JN with the help of ME. The manuscript was drafted by JN, and revised, read and approved in final version by all authors.
